# Second surgery after vertical paramedian hemispherotomy for epilepsy recurrence

**DOI:** 10.1016/j.heliyon.2023.e14326

**Published:** 2023-03-11

**Authors:** Cedric Baltus, Bouchra El M’Kaddem, Susana Ferrao Santos, José Géraldo Ribeiro Vaz, Christian Raftopoulos

**Affiliations:** a Department of Neurosurgery, University Hospital St-Luc, Université Catholique de Louvain, Av. Hippocrate 10, 1200, Brussels, Belgium; b Department of Pediatric Neurology, University Hospital St-Luc, Université Catholique de Louvain, Av. Hippocrate 10, 1200, Brussels, Belgium; c Refractory Epilepsy Center, University Hospital St-Luc, Université Catholique de Louvain, Av. Hippocrate 10, 1200, Brussels, Belgium

**Keywords:** Amygdala, Drug-resistant epilepsy, Hemispherotomy, Paramedian, Rasmussen, Repeat, Vertical, Fail vertical paramedian hemispherotomy

## Abstract

**Background:**

Vertical Paramedian Hemispherotomy (VPH) is considered an effective surgical treatment for drug-resistant epilepsy with 80% of patients experiencing seizure freedom or worthwhile improvement. Identifying persistent connective tracts is challenging in failed VPH.

**Methods:**

We reviewed our series of consecutive patients undergoing VPH for hemispheric drug-resistant epilepsy and included cases with recurrent epileptic seizures undergoing second surgery with at least 6 months of postoperative follow-up. The cases were extensively assessed to propose a targeted complementary resection.

**Results:**

Two children suffering from seizure recurrence following hemispherotomy leading to second surgery were included. After complete assessment, persisting amygdala residue was suspected responsible for the epilepsy recurrence in both patients. Complementary resection of the amygdala residue led to seizure freedom for both patients (Engel IA/ILAE Class 1) without complication. Different diagnostic tools are used to assess patients after failed hemispherotomy including routine EEG, prolonged video EEG, MRI (particularly DTI sequences), SPECT or PET scans and clinical evaluation. These tools allow to rule out epileptic foci in the contralateral hemisphere and to localize a potentially persisting epileptogenic zone. Assessment of these patients should be as systematic and integrated as the initial workup. Although our two patients suffered from Rasmussen's encephalitis, seizure recurrence after VPH has been described in other pathologies.

**Conclusion:**

Lying deep and medially in the surgical corridor of VPH, the amygdala can be incompletely resected and cause recurrent epilepsy. Complementary selective resection of the amygdala residue may safely lead to success in epilepsy control.

## 1 Introduction

Hemispherotomy is considered an effective surgical treatment for drug-resistant epilepsy (DRE) in cases of diffuse hemispheric epileptic activity (including perinatal hypoxia, Rasmussens's encephalitis, cortical dysplasia, Sturge-Weber disease) with overall 80% of patients experiencing seizure freedom or worthwhile improvement [[1], [2], [3]]. Rasmussen's encephalitis (RE), first described in 1958 [4], is a progressive hemispheric epileptic disorder affecting approximately 2 in 10 million aged 18 years and younger per year [5,6]. Authors report significant seizure reduction in the immediate postoperative period for 100% of patients and a long-term seizure freedom in 70–90% of patients with RE undergoing hemispherotomy or its variants [5,[7], [8], [9]]. Disabling seizures persist in a minority of these patients who may be candidates for complementary resection. Santos et al. [10], in 2020, report favorable outcomes of reoperation with acceptable complication rates. We review and discuss the workup and complementary surgical treatment after failed VPH supported by two cases managed in our center.

## 2 Materials and methods

**Fig. 1:**
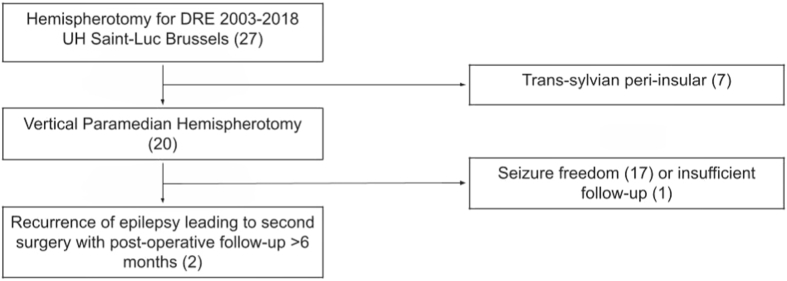
Patients inclusion chart.

We retrospectively reviewed all the patients undergoing vertical paramedian hemispherotomy (VPH) for DRE in our neurosurgical department between 2003 and 2018. All cases were performed by the same senior neurosurgeon (CR). From these patients, we analyzed the ones presenting persisting or recurrence of epileptic seizures. We only included patients with a postoperative follow-up of at least 6 months after the last surgery (Fig. 1). All the included patients were once again discussed at our refractory epilepsy multidisciplinary meeting and extensively assessed to propose a targeted complementary resection. We reviewed demographics, history, clinical presentation, age at first surgery and surgical technique, time to epilepsy recurrence, preoperative workup, age at second surgery and surgical technique, complications and neurological outcome (Table 1).

**Table 1:** Demographics and clinical findings.

	Patient 1 (♀)	Patient 2 (♀)
Age of first seizures	3 years	6 years
Type of seizure	Clonic seizures with vegetative symptomatology and right status epilepticus	Tonic-clonic seizures and right status epilepticus
Etiology	Right Rasmussen's encephalitis	Right Rasmussen's encephalitis
Seizure frequency at first surgery	20 seizures/day	3–10 seizures/day
Age at first surgery	4 years	7 years
First surgery type	Right VPH	Right VPH
Time to recurrence	Immediate	1 month
Type of recurrent seizure	Vegetative symptomatology	Vegetative symptomatology and speech impairment
Seizure frequency at second surgery	25 seizures/day	1 seizure/month
Age at second surgery	8 years	9 years
Second surgery type	Right amygdalectomy	Right amygdalectomy
Epilepsy outcome after second surgery (follow-up)	Engel class IA/ILAE class 1 (12 months)	Engel class IA/ILAE class 1 (21 months)

## 3 Results

Between 2003 and 2018, 27 patients underwent hemispherotomy for refractory epilepsy in our department. The diagnosis was sylvian stroke in 8 cases, Rasmussens's encephalitis in 5 cases, hemimegalencephaly in 4 cases, cortical dysplasia in 3 cases, Sturge-Weber syndrome in 2 cases, sylvian hemorrhage in 2 cases, encephalomalacia in 1 case, West syndrome in 1 case and tumoral scar in 1 case. Twenty patients underwent vertical paramedian hemispherotomy of which 17 were seizure free. One patient had recurrence leading to second surgery which allowed partial improvement of seizures in the immediate post-operative setting (1 week) and was then lost for follow-up abroad. The remaining 2 patients were female, and both suffered from RE. They had persistent or recurrent seizures with predominant vegetative symptomatology following surgery. They had undergone right VPH, respectively at the age of 4 and 7. An extensive assessment led to the hypothesis of persistent amygdala for both patients. They underwent resection of the amygdala residue at the age of 8 and 9. Seizure freedom was observed respectively 12 months and 21 months after complementary resection. There was no need for cerebrospinal fluid shunt nor further surgical procedure.

### 3.1 Patient 1

**Fig. 2:**
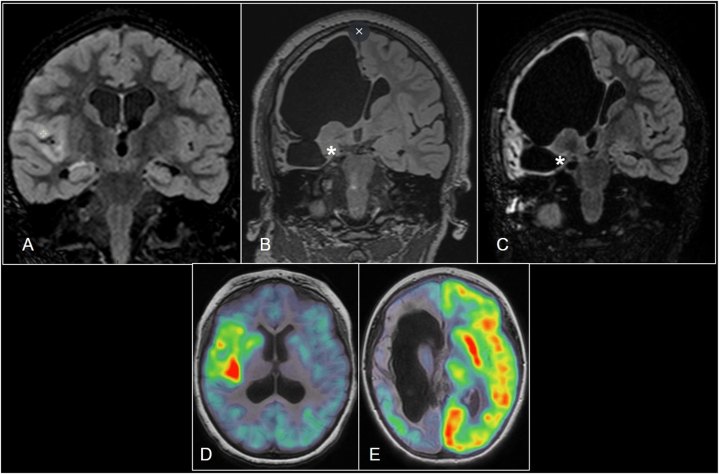
Patient 1 modified coronal sections using FLAIR sequences showing hypersignal in the peri- insular cortex on pre-hemispherotomy MRI (A); possible persisting connection exiting the right amygdala marked by a “*” on pre-amygdalectomy MRI (B); complete resection of right amygdala marked by a “*” on post-amygdalectomy MRI (C); ¹⁸F-fluorodeoxyglucose (¹⁸F-FDG) PET scans fused to T1 weighted-MRI showing pathological hyperactivity in the right peri-insular region on the pre- hemispherotomy imaging (D); resulting hypoactivity in the right peri-insular region compared to the normal activity of the left hemisphere on the pre-amygdalectomy imaging (E).

The first patient was a girl of Moroccan origin with normal and uneventful prenatal history and delivery. Her epilepsy started when she was 3 years old, after a pharyngitis treated by amoxicillin then adenotonsillectomy. Seizures presented with clonic jerks, loss of consciousness and hyper sialorrhea. After 2 months she suffered from status epilepticus in the right hemisphere. She was admitted to the intensive care unit where she was treated with intravenous immunoglobulins along with corticotherapy without significant improvement. She then underwent plasmapheresis which provoked only temporary improvement. She was treated with valproic acid, levetiracetam, carbamazepine, topiramate and lamotrigine. She developed progressive left hemiparesis. Prolonged video-EEG showed continuous right fronto-temporal epileptic activity and MRI revealed T2 and FLAIR hypersignal of the right frontal and temporal insular cortex (Fig. 2A). Positron emission tomography (PET) scan demonstrated right fronto-temporo-insular hyperactivity suggestive of RE (Fig. 2D). She underwent a right VPH aged 4. Histopathology revealed possible RE at early stage (1–2). Clonic seizures immediately ceased, and she was erroneously considered seizure free for 22 months, allowing interruption of anti-seizure medication (ASM). However, early postoperative episodes of vomiting evolved into more frequent and intense vegetative symptomatology with backache, speech impairment, loss of contact, tachypnea, tachycardia, nausea, and occasional vomiting. Carbamazepine and clobazam were reintroduced allowing initial significant improvement. A complete workup was repeated with an electroencephalogram showing quasi-continuous epileptic activity in the right hemisphere, therefore not correlated with the symptoms. There was no independent activity from the left hemisphere. MRI with DTI revealed complete disconnection of the corpus callosum. PET scan was not contributive showing hypometabolism in the right cerebral hemisphere (Fig. 2E). Even though the electrophysiological and radiological workup were poorly contributive, the senior surgeon suspected the responsibility of the amygdala residue (Fig. 2B). The patient underwent resection of that residue (Fig. 2C) at the age of 8 leading to seizure freedom for a total follow-up of 12 months (Engel class IA/ILAE class 1). ASM were interrupted 4 months after surgery and temporarily reintroduced because the patient reported recurrent seizures that were finally considered as psychogenic non-epileptic seizures after extensive work up.

### 3.2 Patient 2

**Fig. 3:**
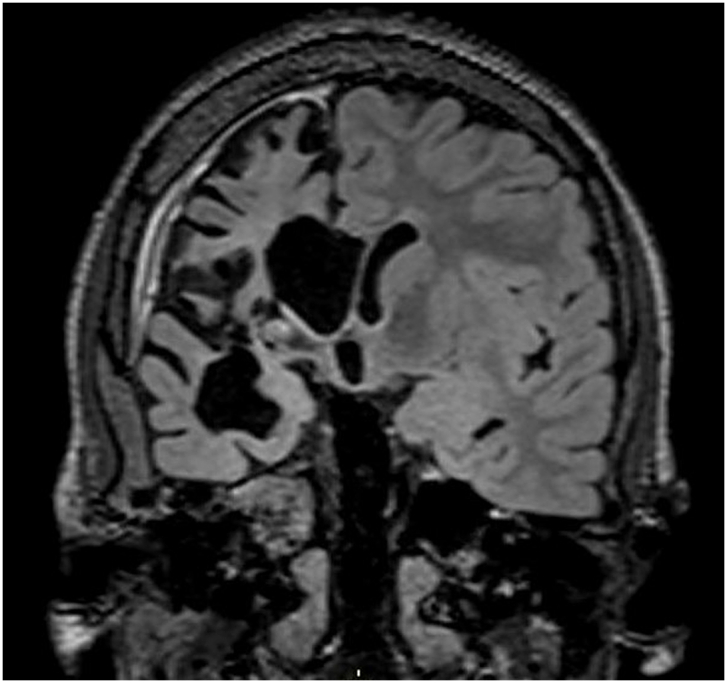
Patient 2 modified coronal sections using FLAIR sequences showing possible persisting connection exiting the right amygdala on pre-amygdalectomy MRI.

The second patient was a Caucasian girl with normal and uneventful prenatal history and delivery. Her epilepsy started with a tonic-clonic seizure followed by right hemispheric status epilepticus at the age of 6 years. Persisting seizures were preceded by palpitations and described as clonic movements of the left hemibody with bradyphrenia and moderate speech impairment. She developed progressive left hemiparesis. She was treated with carbamazepine, levetiracetam and lamotrigine. Prolonged video-EEG showed right hemispheric epileptic activity and MRI revealed diffuse right hemispheric atrophy. PET scan demonstrated hypometabolism in the right cerebral hemisphere. RE was evoked and she was treated with intravenous immunoglobulins (2g/kg) along with corticotherapy without significant improvement. She underwent right VPH at the age of 7 years. Histopathology confirmed RE (stage 3 or 4). She was seizure free for 1 month then showed recurrence of vegetative symptomatology with tonic movements of the upper lip, forced breathing, tachycardia, and mydriasis. Her treatment was adapted for carbamazepine, topiramate and clobazam. A complete workup was repeated with an EEG showing persisting right hemispheric epileptic activity and MRI with DTI revealed complete disconnection of the corpus callosum and the right insula. Again, the amygdala residue was suspected responsible (Fig. 3). She underwent selective right amygdalectomy at the age of 9 leading to seizure freedom for a total follow-up of 21 months (Engel class IA/ILAE class 1) allowing complete interruption of ASM 1 year after surgery. The patient is monitored by her pediatric endocrinologist for central early puberty and is successfully treated with intramuscular Decapeptyl 11,25mg once every 12 weeks. She is also monitored by her pediatric orthopedist for left lumbar scoliosis successfully treated conservatively with a Griffet-Thevenot-Barral (GTB) corset.

## 4 Discussion

**Fig. 4:**
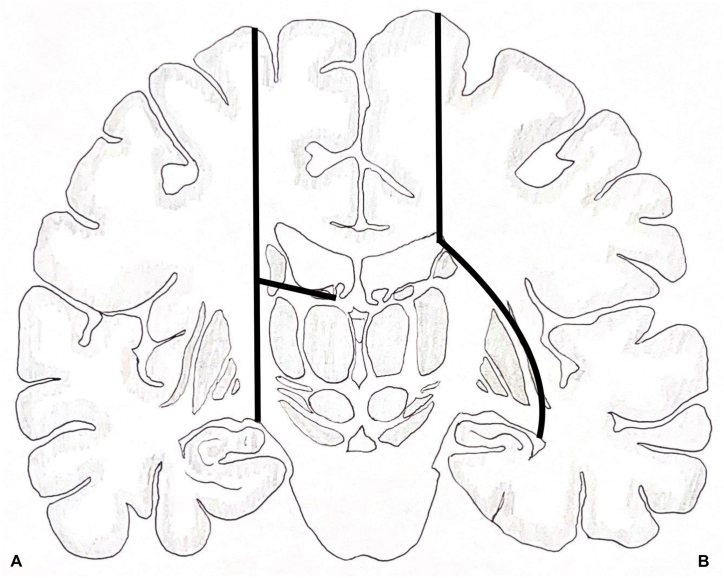
Vertical paramedian hemispherotomy technique described by Delalande (A) and modified technique described by Raftopoulos (B).

Over the past 70 years, resection has slowly been replaced by disconnection surgery for treatment of hemispheric DRE, resulting in the near disappearance of superficial cerebral hemosiderosis [11]. As for most of the specialized teams performing hemispheric resection or disconnection, we have standardized our surgical technique using VPH, modified from its previously well described and illustrated technique [8,11]. Compared to VPH described by Delalande et al. [8], we have decided to perform a pre-central rather than central craniotomy to avoid Trolard's Vein. Then, instead of a central pallium and corpus callosum (CC) disconnection targeting the choroidal fissure, we perform a precentral vertical disconnection of the pallium and truncus of the CC targeting the lateral border of the temporal horn staying lateral to the lenticular nucleus in other words subinsular. Instead of disconnecting the posterior CC, we start by disconnecting the anterior CC including the genu and the rostrum with a particular attention to preservation of the fornix. Instead of a gyrus rectus resection with lateral incision we perform a sub rostrum disconnection including Brodmann Area 25. We continue by disconnecting the splenium of the CC exposing the great cerebral vein of Galen. We then disconnect the floor of the ventricular trigone successively through the crus of the fornix, the fasciolar gyrus (part of the intralimbic gyrus) and the posterior part of the parahippocampal gyrus (part of the limbic gyrus) to reach the ambient cistern. Instead of performing an antero-posterior disconnection lateral to the thalamus (Fig. 4A) we stay lateral to the lenticular nucleus towards the piriform lobe, disconnecting the limen insulae and the amygdala (Fig. 4B). The main objective of these modifications is to keep as much as possible intact the lenticular nucleus which intervenes in numerous activities, especially motor processing. Its connectivity with the thalamic nuclei might play an important role in the contralateral motor recovery following surgery. This is supported by literature focusing on patients recovering from stroke depending on the affected area with indisputable better results when the posterior limb of the internal capsule, adjacent corona radiata, basal ganglia and thalamus are spared [12].

We believe that our modified VPH technique requires a sharp turn at the lower level of the lenticular nucleus to reach the amygdala which is particularly medial in the surgical corridor. Its limited access implies a higher technical difficulty to fully disconnect the amygdala and therefore puts the patient at risk of a remnant potentially responsible for epilepsy recurrence. We identified no specific factor generating a specific difficulty in our two cases.

RE is a well-established indication for hemispherotomy [1,5]. A recent data meta-analysis demonstrated that specifically in RE, a shorter duration of seizure onset to surgery, younger age of onset and younger age at surgery are associated with positive seizure outcome [5]. Young age is therefore not considered a contraindication to surgery [13]. Guan et al. report seizure-freedom for 5/10 patients undergoing reoperative hemispherectomy and 4 others exhibiting 90% reductions [9]. The same authors address whether chronic inflammation and epileptogenic zones remain multifocal in the cortical hemisphere or affect the contralateral hemisphere and that localizing the exact affected cerebral area in RE patients remains a challenge. Considering the invasiveness of such treatment with unavoidable alteration of contralateral motor and visual functions, preoperative hemiparesis and/or visual field deficit frequently encountered in RE can make hemispherotomy an acceptable option for patients and their families as well as the medical team.

Reoperation following failed epilepsy surgery, first described in 1954 [14] has gained increasing interest in the scientific community from the end of the 1990s onwards [15]. Failed epilepsy surgery refers to the persistence of seizures or failure to achieve the expected epilepsy control [1,16]. With safer techniques and more patients treated, multidisciplinary teams are facing the challenge of uncontrolled epilepsy after resective or disconnective surgery.

Repeated surgery after a failed hemispherotomy has been shown to be very effective by multiple teams. Shimizu et al. reported 100% seizure freedom for 3 cases of repeat hemispherotomy in an initial series of 34 patients undergoing hemispherotomy [17]. The same success rate was reported by Mittal et al. in a single case report [18]. Kiehna et al. reported seizure freedom in 5 out of 8 patients (62,5%) undergoing repeat hemispherotomy [19]. In a large series of 36 patients reoperated at the Cleveland Clinic, conversion to anatomical hemispherectomy led to decreased seizure frequency in 45% and seizure freedom in 19% of patients [20]. Girishan et al. recently published an even larger series of 39 patients undergoing endoscopic vertical approach hemispherotomy. Four of the 8 patients with persisting seizure, all of whom showed persistent connections on MRI, underwent complementary endoscopic resection leading to 100% seizure freedom [21]. A case has recently been reported describing postoperative mesio-temporal seizures via amygdalofugal pathway, ceasing after temporal resection [22]. RE is one of the etiologies found in patients with seizure recurrence leading to reoperative hemispherotomy. It represented 14% in a series of 36 patients [20] and 36% in a series of 18 patients [10] with malformation of cortical development being the most represented etiology in these series. In our series, a third patient suffering from RE underwent reoperative hemispherotomy but was excluded for insufficient last follow-up as she left the country directly after the surgery to her homeland. She was followed-up abroad and was not reevaluated postoperatively by our team. The uniformity of the initial etiology is a particular aspect of our series, considering that RE represented 5 patients in a total of 27 patients undergoing hemispherotomy. Due to the small number of cases, we found no explanation to this specificity which may be due to fortuitousness.

There are two main reasons for failed hemispherotomy. The first is the presence of persisting micro-structural connections allowing epileptogenic diffusion in the remaining healthy hemisphere. Authors address particularly 3 diffusion areas: the corpus callosum, the anterior commissure and the hippocampal commissure [1]. The second reason is bi-hemispheric epileptic activity. Differentiating these 2 situations is a crucial part of the workup after a failed hemispherotomy. PET scan is of particular interest in assessing and comparing activity in both hemispheres. It can also help to identify persisting hyperactive tissue in the disconnected hemisphere. MRI with DTI studies is highly effective to identify persisting fiber tracts that could explain persisting seizures. Kiehna et al. also demonstrated in their series that DTI showed limited but definite residual connections between the two hemispheres, in each of the eight cases of failed hemispherotomy, mainly across the rostrum/genu of the corpus callosum [18]. Unfortunately, the profoundly modified neuroanatomy of these patients makes this modality sometimes very difficult to interpret even for experienced and highly specialized neuroradiologists. EEG, including prolonged and video-EEG, is the gold standard and the only modality to confirm the presence of epileptic activity. It should appropriately and extensively be repeated after a failed hemispherotomy. Nevertheless, the pathologic hemisphere is disconnected but left in place during hemispherotomy, an epileptic activity detected in this hemisphere isn't necessarily clinically relevant. Finally, clinical examination and history are key to adequately identify and target the epileptogenic focus and to evaluate the severity of persisting seizures, which is a major criterion in the decision to offer a repeat surgery or not. We faced major difficulties to interpret the clinical presentation of recurring symptomatology after complementary resection for our first patient. We concluded that she presented psychogenic non-epileptic seizures after observing no clinical and electrophysiological differences when ASM were increased.

Specific techniques aiming for higher security and/or better surgical results are described whether used during the first and/or second surgery. In a series of 32 patients undergoing hemispherectomy with intraoperative 3-T MRI with DTI, incomplete disconnection was suggested in 11 patients who underwent immediate additional surgery [23]. Neuronavigation must be used in a standard fashion for the first surgery and especially for the second surgery as the neuroanatomical landmarks may be greatly distorted. Intraoperative scalp EEG on the contralateral hemisphere is used by some teams to detect interictal spikes [1,24,25]. A large prospective observational study published in 2019 showed similar efficacy of endoscopic hemispherotomy compared with open hemispherotomy, with lower levels of blood loss and shorter postoperative hospital stay [26]. One child recently underwent functional hemispherotomy using magnetic resonance imaging–guided laser-induced thermal therapy (MRgLITT) resulting in Engel class IB outcome [27]. This technique under investigation gives hope especially for patients with multiple medical comorbidities.

The retrospective fashion, the low number of cases and the short follow-up are the main limitations of our study which tends to be illustrative and add comprehensive clinical knowledge to the complex field of refractory epilepsy. Heterogeneity of the studies and the lack of standardization in the literature offers little opportunity to define the best generalizable practice.

## 5 Conclusion

Management of failed hemispherotomy is challenging as patients present a profoundly modified anatomy of fiber tracts. We advocate for an extensive clinical, electrophysiological, and radiological workup to identify the persistent connective tracts. Lying deep and medially in the surgical corridor of VPH, the amygdala can be incompletely resected resulting in persistent connections and cause recurrent epilepsy. In our series of consecutive patients who underwent second surgery after failed VPH, the cases with recurrent seizures both suffered from Rasmussen's encephalitis. Both presented seizures with vegetative semiology compatible with an epileptogenic zone within the amygdala, and no other evidence for persistent connective fibers. Complementary selective resection of the residue may safely lead to success in epilepsy control.

## Author contribution statement

All authors listed have significantly contributed to the investigation, development and writing of this article.

## Funding statement

This research did not receive any specific grant from funding agencies in the public, commercial, or not-for-profit sectors.

## Data availability statement

Data included in article/supplementary material/referenced in article.

## Declaration of interest's statement

The authors declare no competing interests.
